# To Contributors and Correspondents

**Published:** 1893-04

**Authors:** 


### TO CONTRIBUTORS AND CORRESPONDENTS.

We would ask special attention to the address of the editorial office of this journal on the front cover.

The editorial and publication offices are distinct and separate, so that matter intended for the former and sent to the latter requires remailing, involving delay.

We hope our friends will kindly remember that all contributions intended for the editor should be sent to 3243 Chestnut Street; exchanges to 716 Filbert Street.

